# Multi-Channel Time-Domain Boring-Vibration-Enhancement Method Using RNN Networks

**DOI:** 10.3390/insects14100817

**Published:** 2023-10-16

**Authors:** Xiaolin Xu, Juhu Li, Huarong Zhang

**Affiliations:** 1 School of Information Science and Technology, Beijing Forestry University, Beijing 100083, China; xuxiaolin1998@bjfu.edu.cn (X.X.); huarong2000@bjfu.edu.cn (H.Z.); 2 Engineering Research Center for Forestry-Oriented Intelligent Information Processing of National Forestry and Grassland Administration, Beijing 100083, China

**Keywords:** beamforming, multi-channel, boring vibration signal, self-attention mechanism, denoising

## Abstract

**Simple Summary:**

Tree trunk damage can be influenced by multiple factors, among which trunk-boring insect infestation plays a significant role. Early external detection of such damage poses challenges. Manual observation remains the prevailing method for controlling tree trunk pests, but it demands a substantial workforce and yields limited outcomes. To address these limitations, acoustic technology has gained popularity, using vibration probes embedded in tree trunks to capture vibrations produced by insect larvae feeding, thereby facilitating the detection of pest larvae. However, traditional methods primarily rely on single-channel vibration signal acquisition, often assuming the proximity of the vibration probe to the sound source. Nevertheless, when the probe’s position exceeds a certain distance from the original, capturing effective drilling vibration signals becomes difficult due to noise interference and other factors. To overcome this constraint, we have developed a novel multi-channel drilling vibration signal acquisition board that enables the distribution of multiple vibration probes at different locations on the tree trunk, allowing simultaneous collection of vibration signals from diverse probes. Additionally, we have devised a multi-channel signal separation model based on attention mechanisms, which effectively denoises and recovers clean target signals from noisy recordings. Experimental results demonstrate that this approach significantly enhances the detection efficiency of trunk-boring insects.

**Abstract:**

The larvae of certain wood-boring beetles typically inhabit the interior of trees and feed on the wood, leaving almost no external traces during the early stages of infestation. Acoustic techniques are commonly employed to detect the vibrations produced by these larvae while they feed on wood, significantly increasing detection efficiency compared to traditional methods. However, this method’s accuracy is greatly affected by environmental noise interference. To address the impact of environmental noise, this paper introduces a signal separation system based on a multi-channel attention mechanism. The system utilizes multiple sensors to receive wood-boring vibration signals and employs the attention mechanism to adjust the weights of relevant channels. By utilizing beamforming techniques, the system successfully removes noise from the wood-boring vibration signals and separates the clean wood-boring vibration signals from the noisy ones. The data used in this study were collected from both field and laboratory environments, ensuring the authenticity of the dataset. Experimental results demonstrate that this system can efficiently separate the wood-boring vibration signals from the mixed noisy signals.

## 1. Introduction

Trees face significant threats from climate change, wildfires, and pest infestations [1]. Various forest disasters, including wildfires, climate change, human encroachment, and tree pathogens and pests, have varying degrees of impact on forest ecosystems, influencing agriculture, forestry, and human livelihood [2]. Among forest pests, wood-boring beetles pose a particularly significant threat, including species such as Agrilus planipennis (Coleoptera: Buprestidae), Semanotus bifasciatus (Coleoptera: Cerambycidae), and Eucryptorrhynchus brandti (Coleoptera: Curculionidae). The larvae of these beetles primarily inhabit the cambium layer of trees, feeding on the wood and disrupting nutrient transportation, leading to the weakening and eventual death of the tree. Unfortunately, infestations by wood-boring beetles are often not easily detected until visible signs of withering or damage on the tree branches appear [3]. A novel approach currently under exploration involves analyzing acoustic vibration signals to detect the presence of insect larvae within tree trunks. This method utilizes a piezoelectric sensor, such as an ICP (integrated circuit piezoelectric) sensor, to convert mechanical vibrations into electrical signals. The collected vibration signals are then inputted into a pre-trained model to detect larva presence and determine whether the tree is infected or not [4]. Despite some studies on wood-boring insect larva detection [5,6], most of these studies rely on a single vibration sensor to receive the signals, making them susceptible to environmental noise that can impact larva detection in outdoor environments. To improve the recognition rate of larvae, we propose using multiple vibration sensors to capture and feed the vibration signals into the model. The primary focus of this work is to preprocess the monotonous vibration signals emitted by the larvae, separating them from signals contaminated by various environmental noise sources that could affect recognition performance. The aim is to isolate and preserve the clean wood-boring vibration signals associated with the insect larvae.

Some studies have applied the technology mentioned above to identify wood-boring vibrations. For example, Alexander Sutin [4] utilized a piezoelectric sensor and a dual-mode charge sensor to record the vibration signals of infected trees. They developed an automatic detection algorithm that determines the presence of larvae based on a threshold of the average pulse rate. In this study, representative feeding sounds of larvae were selected by human experts. Features were extracted from these sounds, followed by binary or multi-class classification analysis in the time, frequency, or scale domains. Ultimately, an automated insect detection system was achieved by optimizing the parameters of radial basis function (RBF) kernels and polynomial kernels. Le Conte et al. proposed using acoustic emission monitoring technology to detect wood-boring insects in wooden cultural heritage instruments preserved in various European museums [7]. The study employed robust data processing based on orthogonal linear transforms, followed by applying the processed signals to distinguish insect signals from environmental noise. Using acoustic detection technology, the study successfully detected larvae measuring approximately 1–2 mm in length within the musical instruments. In addition, in another study, ref. [8] proposed a unified framework for the automated bioacoustic recognition of specific pests.

In natural environments, natural and non-natural noises are typically concurrent. For this study, we categorize all signals unrelated to boring vibration signals as noise signals. As previously demonstrated in the literature [9], the impact of environmental noise on the recognition of boring vibration signals has been confirmed. Traditional multi-channel denoising algorithms include adaptive filtering [10], post-processing Wiener filtering [11], and spatial noise suppression techniques [12]. However, these methods often require prior knowledge or substantial computational resources. The separation and denoising results of these methods may only be partially satisfactory. With the continuous development of acoustic technology, applying emerging techniques to boring vibration signals has become increasingly feasible.

Furthermore, most previous studies have primarily focused on single-channel analysis [13]. Indeed, in the context of detecting wood-boring insects using acoustic techniques [14,15], using multiple sensors often offers significant advantages compared to a single sensor.

Multi-channel boring-vibration signal-separation techniques benefit from using data received from multiple vibration sensors, enabling them to acquire vast information. The more input information we have, the more information we can extract. Furthermore, from a traditional perspective, multi-channel analysis enables beamforming, which exhibits strong generalization and robustness [16]. Research on multi-channel end-to-end speech separation primarily focuses on two directions: neural network beamforming and extending single-channel models to multi-channel settings. The output-based neural network beamforming methods mainly include DeepBeam and Beam-TasNet approaches. DeepBeam utilizes time-domain multi-channel Wiener filtering. It starts by selecting a reference microphone and employs a single-channel enhancement network trained to enhance the signal from that particular microphone. This enhanced signal is then utilized as the target for Wiener filtering, aiming to obtain the optimal filter parameters for the remaining vibration sensor. The objective is to obtain a cleaner speech signal through this process [17]. In the Beam-TasNet method [18], a combination of time-domain and frequency-domain approaches is employed. A multi-channel TasNet performs an initial separation of the mixed speech signals, resulting in preliminary separated speech. Subsequently, the MVDR (minimum variance distortionless response) weights are estimated using the separated speech as references in the frequency domain. These weights are then applied to the mixed speech to obtain the final separated speech. Beam-TasNet incorporates time-domain techniques for phase estimation and utilizes spatial features to achieve the desired speech separation. In addition to DeepBeam and Beam-TasNet, another significant work in neural network-based beamforming is the Filter-and-sum Network (FaSNet) [19]. Indeed, FaSNet emphasizes information sharing among multiple channels to optimize time-domain filters jointly. There have been significant advancements in the development of insect sound detection technology [20], with many mature methods available. These methods involve the application of various vibration sensors in different substrates and employ signal processing techniques. Therefore, applying multi-channel speech enhancement and separation techniques to boring vibration signals using similar processing techniques is feasible.

With the advancements in deep learning and neural network technologies [21], acoustic technology has also experienced further development in this regard. Combining deep learning with speech enhancement and separation techniques has further propelled the growth of wood-boring larva detection. Rigakis et al. [15] proposed an automated system called TreeVibes, which collects vibration sounds and converts them into analyzable data. The data are then fed into a deep learning model, such as the Xception model, for analysis to determine whether the trees are affected by insect infestation. Compared to traditional manual detection methods, this approach significantly increases the chances of early detection of insect infestations. Mankin et al. [22] proposed using acoustic technology for detecting and monitoring insect pests. They collected vibration data from within tree trunks and analyzed them using deep learning models to determine the presence of pests.

In addition, a novel approach called the attention mechanism has emerged quietly. The attention mechanism enables the model to focus on different parts of the input sequence while generating the output sequence. It enhances the weights assigned to the relevant features and disregards irrelevant portions of the input sequence. By employing multiple iterations of learning linear projections, the model can attend to different representation subspaces at different positions [23]. The attention mechanism has been widely adopted in various domains, such as image recognition [24], audio processing, sentiment analysis [25], and more.

This study takes the boring vibrations of emerald ash borer (EAB) larvae as the research subject. The larvae of the emerald ash borer (EAB) indeed have a significant impact on ecological environments [26], particularly in terms of the damage they inflict on white ash trees. Traditional methods for identifying wood-boring insect infestations include manual observations [27] and using pheromones [28]. Manual identification involves visually inspecting the presence of “D-shaped” exit holes on the tree bark and checking for live larvae inside the tree. Indeed, this method is labor-intensive and less effective in achieving efficient pest control. Its limitations make obtaining satisfactory results in terms of prevention and control complex. Using vibration signals emitted by larvae during their activity and feeding as a clue to examine the presence of EAB larvae inside tree trunks is an effective method that saves significant human resources. Therefore, this paper proposes an end-to-end multi-channel boring-vibration signal-separation model based on attention mechanisms. Our team collected and synthesized all the data used in this study. This includes clean boring vibration signals and synthetic signals generated through simulations. Our results demonstrate that our proposed model effectively suppresses noise compared to single-channel and multi-channel models. In the case of utilizing different numbers of vibration sensors and networks, our model has demonstrated improvements in SNR and SDR ranging from 5% to 15%.

## 2. Collection and Preprocessing of Dataset

### 2.1. Field Data Collection

**Figure 1:**
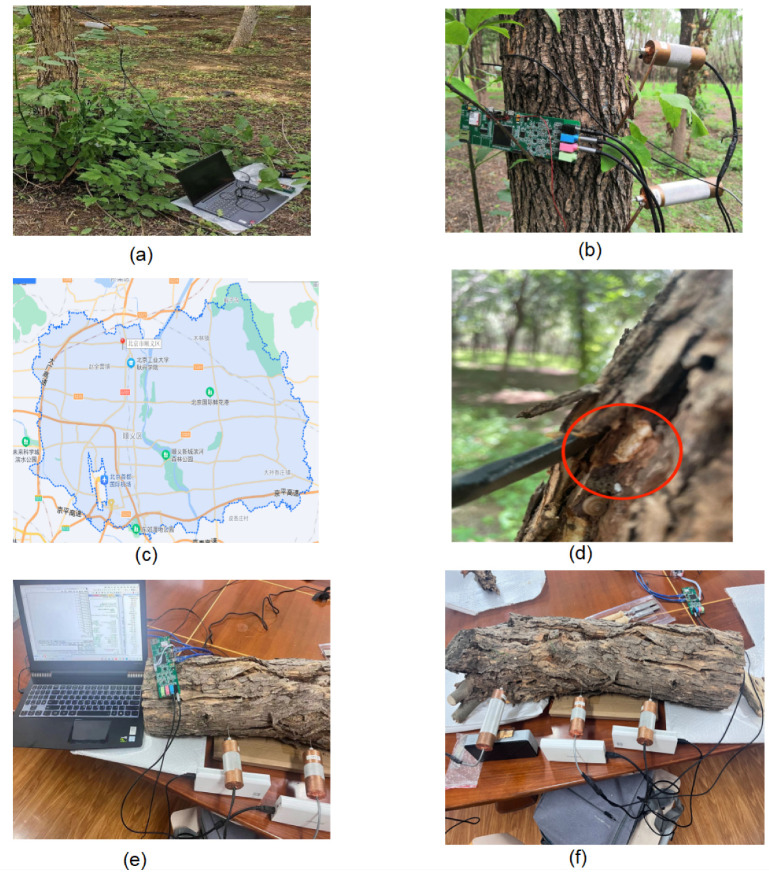
(**a**,**b**) depict data collection work in the field environment, utilizing four probes for data acquisition. (**c**) represents the specific location of data collection and sample collection in outdoor environment. (**d**) illustrates larvae found beneath the bark surface after collecting data from trees in the field environment. (**e**,**f**) represent data collection using a data acquisition board in a laboratory setting.

We selected multiple locations for collecting drill string vibration signals in outdoor environments, including Shunyi District and Changping District in Beijing. Probes were inserted into the tree trunks approximately 3–5 cm below the surface to detect the presence of target drilling vibration signals. As shown in Figure 1a, a 40-cm segment was selected from the target tree where the drilling vibration signal is relatively active. Four probes were inserted at a radius of 10 cm, and data were collected using a data acquisition board and saved on an SD card. Each recording session lasted approximately 5 h, resulting in over 30 h of field data collection. To ensure the practicality of the data, we conducted some minor preprocessing on the field data. These data will be applied in experiments conducted in real-world environmental conditions to validate the separation performance of the models proposed in this study. In these experiments, four vibration sensors were used for data collection.

### 2.2. Data Collection and Filtering

Regarding the enhanced data collection part, we first selected a certain quantity of white ash trees from the field. These trees included three types: infected but still alive, dying from infection, and uninfected trees. The collected segments of these white ash trees were trimmed to a fixed length, and all small branches and leaves on the tree trunks were removed. As shown in Figure 1. All procedures were conducted in a controlled environment in an unmanned laboratory to collect clean wood-boring vibration signals. Beijing Forestry University and Beihang University (Beijing University of Aeronautics and Astronautics) jointly developed the vibrating sensor for collecting vibration signals. The recorded wood-boring vibration signals were sampled at 44.1 KHz with a bit depth of 16 bits. Our research team independently developed the multi-channel drilling vibration signal acquisition board for data collection. The collected data will be stored on an SD card, which can be read and processed using a computer. Due to the highly faint nature of wood-boring vibration signals, the data collection process was conducted in an unmanned laboratory. Before recording, experts used headphones to listen and ensure the presence of larval activity in the sampled tree segments. After ensuring that the larvae were relatively active within the tree trunk, the wood-boring vibration signals from that tree segment were collected. For tree segments with relatively active larval activity, audio components of approximately 10 h were recorded. In the case of tree trunks nearing death or already dead, the larval activity inside was weak or almost non-existent, resulting in the collected signals predominantly consisting of instrument noise.

The enhanced wood-boring vibration signals from laboratory and outdoor environments need further processing. This involves removing noise generated by manual operations at the start and end of audio segments, including footsteps and headphone-related noise. Abnormal or unnecessary noise in spectrograms will also be manually removed, along with segments featuring extended periods of no larval activity in outdoor recordings. We have applied trimming to these signals to ensure their effective utilization.

Regarding the selection of environmental noise, we collect it using the same configuration as for capturing the wood-boring vibration signals from the larvae.

### 2.3. Dataset Production

For our multi-channel wood-boring-vibration signal-separation model, processed clean wood-boring vibration signals must undergo synthesis processing. The training dataset mainly consists of two components. One part involves laboratory simulation and synthesis, accounting for 20% of the dataset, primarily aimed at validating the impact of source location on signal acquisition. The other part comprises four-channel signals collected under laboratory conditions using a multi-channel data acquisition board, necessitating the addition of noise data to enhance features. The noise datasets are sourced from environmental noise contained in natural outdoor environments. Additionally, we can randomly adjust the signal-to-noise ratio to improve the model’s training generalization. In the experiment, 90% of the generated dataset is allocated as the training set, with the remaining portion used as the test set. Due to hardware constraints, our training set consists of 3500 segments, while the test set contains 200. To facilitate readers to understand the paper more deeply, we have selected some vibration signal data collected and put them in the Supplementary Materials for reference.

## 3. Data Acquisition Equipment

### 3.1. Introduction to the Principle of Vibration Sensors

**Figure 2:**
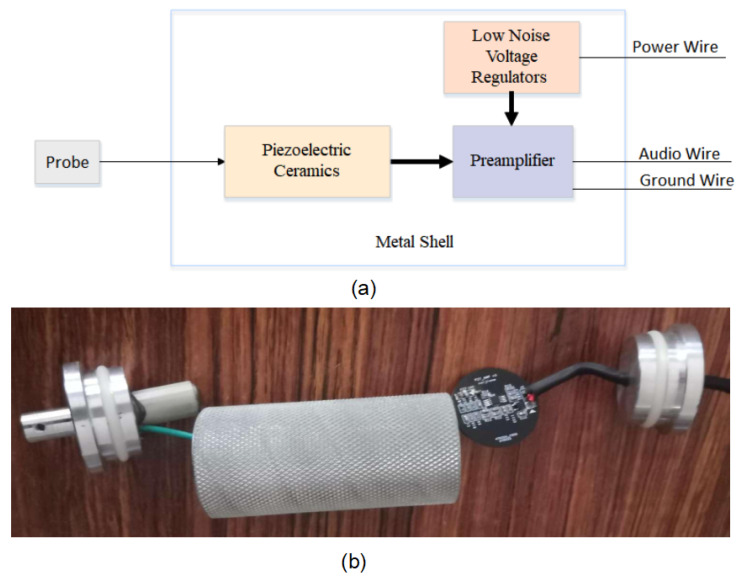
Physical representation and schematic design of the vibration sensors. (**a**) represents the schematic diagram of the Vibration Sensors, (**b**) displays the physical representation.

The vibration sensors are sealed inside a tubular metal casing to adapt to the forest environment. It includes the piezoelectric module, the preamplifier, and the low-noise voltage regulators. The piezoelectric module consists of four parts: a probe, a base, a piezoelectric material, and a mass block, used to convert the vibration excited by boreholes into electrical signals. The probe is threaded and embedded into the tree trunk, and vibration is transmitted to the mass block and piezoelectric material through the probe and base. To improve sensing sensitivity, the piezoelectric material adopts four columnar axially polarized lead zirconate titanate piezoelectric ceramics (PZT) in series, with a sensitivity of 500 mV/g. The preamplifier proportionally amplifies the weak voltage generated by the piezoelectric module, converting the weak high-impedance raw signal into a low-impedance voltage signal that is easy to transmit for subsequent measurement. Using an in-phase amplifier with a voltage amplification factor of 40 dB The low noise voltage regulators can automatically adjust the output voltage to stabilize the fluctuating power supply voltage within the set value range, enabling various circuits or electrical equipment to operate normally at the rated voltage. External capacitors (usually 10 nF) must be connected to minimize noise voltage. The input voltage is about 5 V, the output voltage is everywhere 4.7 V, and a voltage drop of back 0.3 V is used to operate the voltage regulator. The function of the metal shell is to shield external interference with the vibration sensor. Many complex interference sources, such as external electric and magnetic fields, cannot be seen or touched in the outdoor working environment. After adding a shielding cover to the sensor collection device, it can effectively isolate these external interference sources.As shown in Figure 2, both the schematic diagram and the physical image of the Vibration Sensors are presented.

### 3.2. Introduction to the Principle of Vibration Signal Acquisition Board

**Figure 3:**
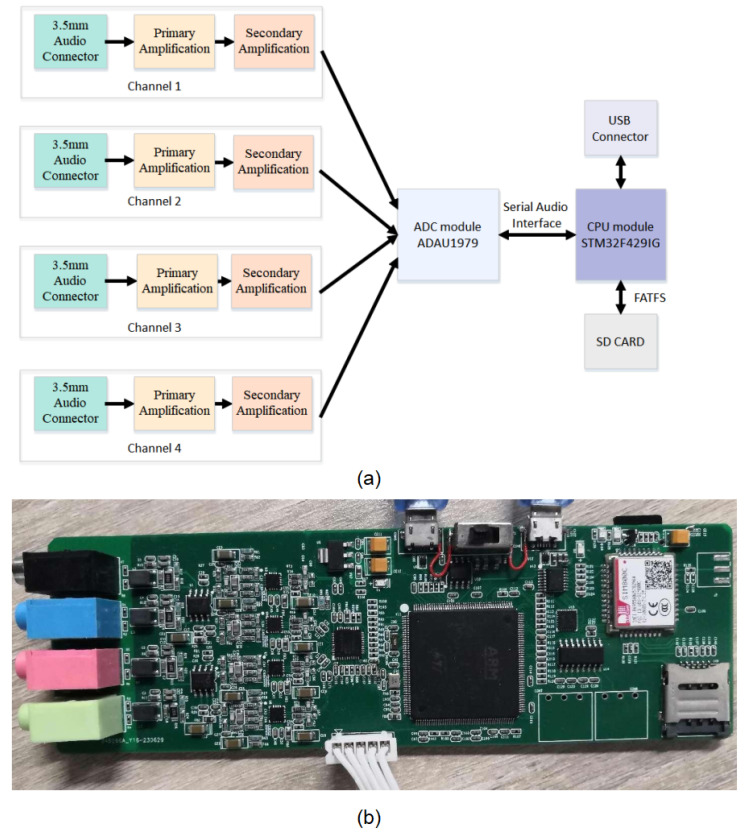
(**a**) represents the schematic diagram of the data acquisition board, (**b**) displays the physical representation.

The acquisition board is composed of four parts: a primary amplification module, a secondary amplification module, an ADC conversion module, and a CPU acquisition and storage module. The primary amplification circuit mainly serves as a voltage follower, converting high-output impedance AC vibration signals into low-output impedance AC vibration signals through impedance transformation. The function of the secondary amplification circuit is to convert the vibration signal at a single end into a bipolar vibration signal. In addition, the vibration signal is filtered to remove signals below 100 Hz and above 15 KHz. According to research, the frequency of the vibration signal of borer pests is between 6 KHz and 12 KHz. The two-stage amplification circuit simultaneously processes four vibration signals. The function of the ADC module is to convert analog vibration signals into digital vibration signals. The ADAU1979 IC is used for completing the process, with a sampling rate of 44.1 KHz and sampling bits of 16 bits. At the same time, it collects four vibration signals and dynamically adjusts the gain of each channel through parameter settings. The data are sent to the CPU through the SAI interface. The function of the CPU module is to receive digital audio signals, perform FATFS format conversion, and then store them in a given file based on different channels. The input voltage of the entire acquisition board is 5 V, and the current consumption is 100 mA. After long-term recording and testing, there is no data loss. As shown in Figure 3, both the schematic diagram and the physical image of the acquisition board are presented.

## 4. MultiSAMS Model

### 4.1. Drill String Vibration Signal Enhancement Model

Recurrent neural networks (RNNs) have achieved remarkable success in various speech signal processing tasks in recent years, such as speech recognition, speech synthesis, speech enhancement, and speech separation [29]. To effectively utilize contextual information, we have chosen to use recurrent neural networks (RNNs), which are well-suited for capturing sequential dependencies [30]. Because we need to use multiple vibration sensors in data collection, we have opted to employ time-domain beamforming techniques. While time-domain methods may have some performance differences in robustness compared to frequency-domain methods [31], they offer faster response times and relatively smaller model sizes, requiring fewer computational resources. Based on the abovementioned techniques, we propose a novel approach called MultiSAMS, a time-domain-based multi-channel separation network. And on this basis, we incorporate the multi-head self-attention mechanism module (MSAM), also known as the attention mechanism module. Unlike other methods, MultiSAMS replaces the traditional filtering module with a bidirectional RNN neural network. RNN neural networks are well suited for audio tasks as they effectively capture long-term dependencies.

In MultiSAMS, we also use a one-dimensional convolutional layer to extract data features from each channel, where the size of the one-dimensional kernel is variable and determined by the sum of the context length and window size. Moreover, we calculate the cosine similarity between channels to extract the NCC (normalized cross-correlation) feature. These two features are then concatenated and fed into the MSAM module.

The attention mechanism in the MSAM module provides several advantages. It enables the model to focus more on different channels’ information during the learning process, thus learning the correlations between other channels. By using the attention mechanism, the model can automatically adjust the weights between channels, giving more attention to crucial information and enhancing the model’s performance.

Finally, the concatenated features are passed through the filtering module to obtain the final output. The proposed MultiSAMS method effectively combines the power of bidirectional RNNs, one-dimensional convolutional layers, and attention mechanisms, making it well suited for multi-channel audio signal separation tasks.

Specifically, our beamforming technique is the estimation of time-domain beamforming filters for microphone arrays with N≥2 vibration sensor. We select one vibration sensor as the reference sensor and sum the filtered signals from all vibration sensor channels to better estimate the vibration chosen sensor. We need to divide the signal $x^{i}$ from the vibration sensor into *M* sample frames, with each sample frame having a hop size of $M\in \left[0,\;L-1\right]$.

(1) $$\;x_{t}^{i}=x^{i}\left[tM:tM+l-1\right],\;t\in Z,i=1,\ldots ,N$$

In this equation, *t* represents the index of the frame, and *i* represents the index of the vibration sensor. The operation $x\left[a:b\right]$ selects the values of the vector *x* from index *a* to index *b*.

(2) $${\hat{y}}_{t}=\sum_{i=1}^{N}z_{t}^{i}⊗{\hat{x}}_{t}^{i}$$

In this context, ${\hat{y}}_{t}\in R^{1\times L}$ refers to the beamforming signal at frame *t*, while ${\hat{x}}_{t}^{i}=x\left[tM-L:tM+2L-1\right]\in R^{2L\;+\;1}$ represents the context window around the vibration sensor *i*. The variable $z_{t}^{i}\in R^{2L+1}$ represents the beamforming filter that the microphone *i* is learning, and ⊗ represents the convolution operation. Zero-padding is applied to the context window to ensure that the model has a span of ±L samples across microphone delays.

For the reference microphone, assuming the first microphone is labeled as 1, the input signal would be $x^{1}$, which includes the current frame and *L* past and future samples. For the other microphones, *i*, the signal corresponding to the frame is extracted as follows: $x^{i}=x^{i}\left[tH:tH+L-1\right]$. To be specific, let ${\hat{x}}^{1}\in R^{1\times 3L}$ be the context window of the signal in the reference microphone and ${\hat{x}}^{i}\in R^{1\times L},i=2,\ldots ,N$. To mitigate the impact of other frequency-domain beamforming tasks, we utilize normalized cross-correlation (NCC) as the inter-channel feature [32]. We compute the cosine similarity between $x^{1}$ and $x^{i}$.

(3) $$\left\{\begin{array}{c} {\hat{x}}_{g}^{1}={\hat{x}}^{1}\left[g:g+L-1\right]\\ h_{g}^{i}=\frac{{\hat{x}}_{g}^{1}{\left(x^{i}\right)}^{T}}{{∥{\hat{x}}_{g}^{1}∥}_{2}{∥x^{i}∥}_{2}} \end{array}\right.,\;g=1,\ldots ,2L+1$$

where $h^{}\in R^{1\times \left(2L+1\right)}$ represents the cosine similarity between the reference microphone and microphone *i*, where $f_{g}^{i}$ is a vector of length 2L+1. By averaging the N−1 NCC vectors $f^{i}$ from all other vibration sensors, we obtain the average feature:

(4) $${\bar{f}}^{i}=\frac{1}{N-1}\sum_{i=2}^{N}f^{i}$$

For a specific channel, the input is the center frame of the signal in the reference microphone, denoted as $x^{1}$, which is an $x_{1}$ dimensional vector. A linear layer is applied to $x_{1}$ to create a K-dimensional embedding, represented as Z1. This is accomplished using a weight matrix *D* of size K×L.

(5) $$Z^{1}=X^{1}D$$

where $D\in R^{L\times K}$ is the weight matrix. Subsequently, it is passed to an RNN neural network with a gated output layer, generating C beamforming filters, where C is the number of sources of interest.

(6) $$Q_{1}^{1},\ldots ,k=h_{1}\left(\left|Z^{1},\bar{f}\right|\right)$$

(7) $$e_{c}^{1}=\tanh\left(Q_{C}^{1}O^{1}+b^{1}\right)⊙\partial \left(Q_{C}^{1}X^{1}+q^{1}\right)$$

The mapping function $H_{1}$ is used to generate the output $P_{C}^{1}$, which is then convolved with $x_{1}$ to generate the beamforming output ${\bar{Y}}_{C}^{1}$ for the reference microphone.

(8) $${\bar{Y}}_{C}^{1}={\bar{X}}^{1}⊗h_{c}^{1}$$

For the remaining channels, the beamforming filters $h_{c}^{i},i=2,\ldots ,N$ are estimated. The estimated clean vibration signal from the reference vibration sensor is used as a cue for all remaining vibration sensors for the sources of interest. Firstly, the aforementioned process is applied to compute all NCC features.

(9) $$\left\{\begin{array}{c} {\bar{x}}_{g}^{y}=\bar{x}\left[g:g+L-1\right]\\ g_{c,g}^{i}=\frac{{\hat{x}}_{g}^{i}{\left({\hat{y}}_{c}^{1}\right)}^{T}}{{∥{\hat{x}}_{g}^{i}∥}_{2}{∥{\hat{y}}_{c}^{1}∥}_{2}} \end{array}\right.$$

The filters are convolved, and the outputs of all filters are weighted and summed to obtain the final beamforming output.

(10) $${\hat{y}}_{c}^{}=y_{c}^{1}+\sum_{i=2}^{N}{\hat{x}}^{i}⊗h_{c}^{i}$$

**Figure 4:**
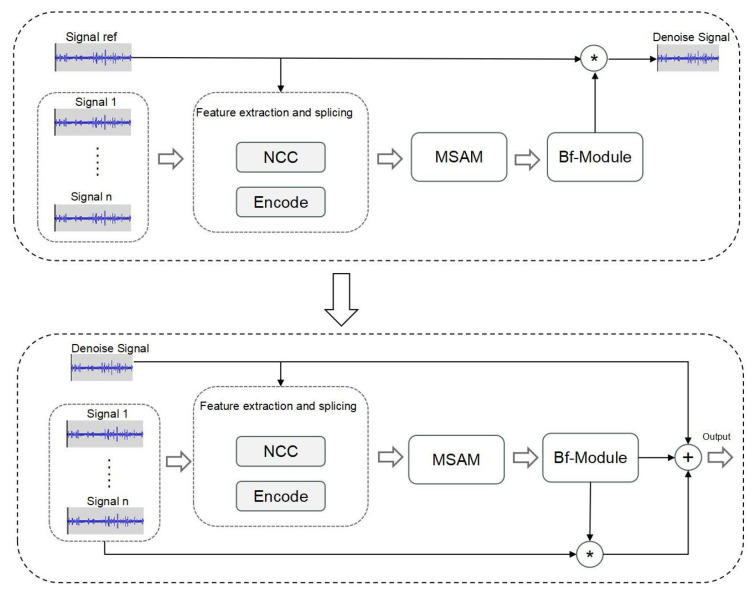
The MultiSAMS architecture includes an “encode” block for extracting data features from each channel using a one-dimensional convolutional network, an “NCC” block for calculating features between channels using normalized cross-correlation, and an attention mechanism module for feature optimization, followed by the beamforming filter module for final processing.

**Figure 5:**
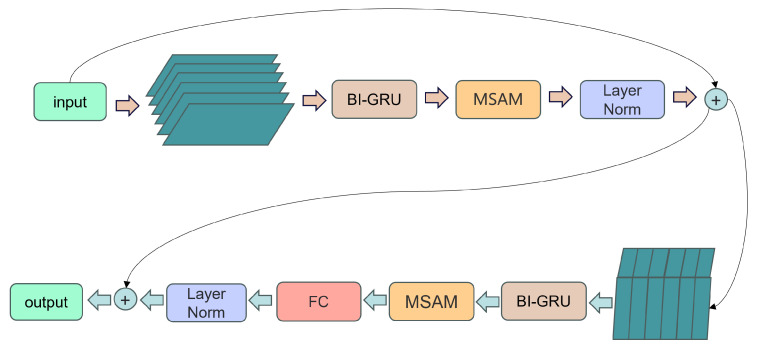
The proposed structure is the beamforming module, which utilizes a multi-layer BI-GRU to model and process sequential data. The sequential data are split into columns and blocks, and features are extracted. Subsequently, a multi-layer MSAM is applied to optimize the extracted features for further processing.

Furthermore, the overall architecture of the MultiSAMS model is illustrated in Figure 4, and the filter-and-sum network (BF-Module) is shown in Figure 5.

### 4.2. Filter-and-Sum Network

Inspired by the work of Luo et al. [33], we utilize neural network modules to enable end-to-end training, replacing traditional beamforming filters. This approach addresses the limitation of fixed operations in traditional filters, allowing for real-time adjustments. We can effectively handle longer vibration signals by employing bidirectional recurrent neural networks (RNNs). Bidirectional RNN networks combine local and global modeling, significantly reducing the computational burden of RNNs and improving efficiency. Moreover, the structure of bidirectional RNNs is relatively simple, making them easy to implement. For an input sequence $V\in R^{M\times L}$, where *M* represents the feature dimension and *L* represents the number of time steps. The input sequence is divided into blocks of length *K* with a stride size of *P*, resulting in *S* equal-sized blocks. These blocks are then concatenated together to form a three-dimensional tensor. The segmentation output *Z* is then passed to the stack of *B* RNN blocks. Each module transforms the input three-dimensional tensor into another tensor of the same shape, containing two sub-modules for intra-block and inter-block processing. The intra-block RNN is bidirectional and applied to the second dimension of the input tensor for each of the *S* blocks. The input tensor for each module is denoted as $Z_{b}$, where *b* represents the block number ranging from 1 to *B*. The shape of the input tensor $Z_{b}$ is $Z_{b}\in R^{M\times K\times S}$, where *M* is the feature dimension, *K* is the block length, and *S* is the number of blocks.

(11) $$W_{b}=\left[l_{b}\left(T_{b}\left[:,:,i\right]\right),i=1,\ldots ,S\right]$$

where $W_{b}\in R^{H\times K\times S}$ is the output of the RNN, $l_{b}\left(•\right)$ is the mapping function, and $T_{b}\in R^{N\times K}$ is the sequence defined by chunk *i*.

Regarding the use of the multi-head attention mechanism, we apply it after the RNN layer by transforming the output $W_{b}$ and feeding it into the attention mechanism to obtain the new output $H_{b}$. The shape of the output $H_{b}$ is then transformed back to its original dimensions to obtain the new $W_{b}$. The specific implementation of the attention mechanism will be described in the next paragraph of the paper.

(12) $${\hat{W}}_{b}=\left[JW_{b}\left[:,:,i\right]+x,i=1,\ldots ,S\right]$$

The fully connected layer aims to restore the feature dimension N. ${\hat{W}}_{b}\in R^{H\times K\times S}$ represents the restored features after this layer. $J\in R^{N\times H}$ and $x\in R^{N\times 1}$ are the weight and bias of the FC layer. In addition, $W_{b}\left[:,:,i\right]\in R^{H\times K}$ represents chunk *i* in $W_{b}$. The input ${\hat{W}}_{b}$ application layer is normalized to obtain the output $LN({\hat{W}}_{b})$. We then perform the residual connection by connecting $T_{b}$ to the original input $LN({\hat{W}}_{b})$, resulting in a new output ${\hat{T}}_{b}$.

(13) $$K_{b}=[e_{b}({\overset{⌢}{T}}_{b}\left[:,i,:\right]),i=1,\ldots ,K]\;$$

The output $K_{b}\in R^{H\times K\times S}$ obtained from the previous step is used as the input to the next RNN block, where the inter-block RNN captures global information. The output of the inter-block RNN is obtained by applying the mapping function $e_{b}\left(•\right)$. The sequence ${\overset{⌢}{T}}_{b}\left[:,i,:\right]\in R^{M\times S}$ is defined by the *i* time step in all *S* blocks. Similar to the previous step’s output *T_b_*, the *K_b_* is also subjected to an attention mechanism layer, a fully connected layer, and a normalization layer. Finally, a residual connection is added to form the output.

### 4.3. Self-Attention Mechanism

The attention mechanism can be described as mapping a query and a set of key-value pairs to an output, where the query, keys, values, and output are all vectors. The output is computed as a weighted sum of the values, and an attention function calculates the weights. In our model, each output at each time step depends on the previous time step. When using the attention layer for feature extraction, all keys, values, and queries originate from the same source. In this scenario, each position can attend to all positions in the preceding layer. This property helps address the issue of long-term dependencies.

In practice, the attention function is computed for queries packed into a matrix *Q*. Similarly, the keys and values are loaded into matrices *K* and *V*. Respectively, the output matrix is then computed as follows:

(14) $$Attention\left(Q,K,V\right)=soft\max\left(\frac{QK^{T}}{\sqrt{d_{k}}}\right)V$$

Linear projections are applied to the queries, keys, and values *h* times, with each projection having its own learned parameters and resulting in different dimensions (dk, dk, and dv, respectively). The attention function is then applied independently to each projection, producing output values of dimension dv. These output values are concatenated together. Finally, the concatenated values are projected again to obtain the final output. This design enables the model to attend to information from different representation subspaces at different positions, addressing the issue of suppression that arises when using a single attention head that averages across different sources of information.

(15) $$MultiHead\left(Q,K,V\right)=Concat\left(head1,\ldots ,headh\right)W^{O}\;$$

(16) $$head_{i}=Attention\left(QW_{i}^{Q},KW_{i}^{K},VW_{i}^{V}\right)$$

where the projections are parameter matrices $W_{i}^{Q}\in R^{d_{\mathrm{model}}\times d_{k}}$, $W_{i}^{K}\in R^{d_{\mathrm{model}}\times d_{k}}$, $W_{i}^{V}\in R^{d_{\mathrm{model}}\times d_{v}}$ and $W_{}^{O}\in R^{d_{\mathrm{model}}\times hd_{v}}$.

**Figure 6:**
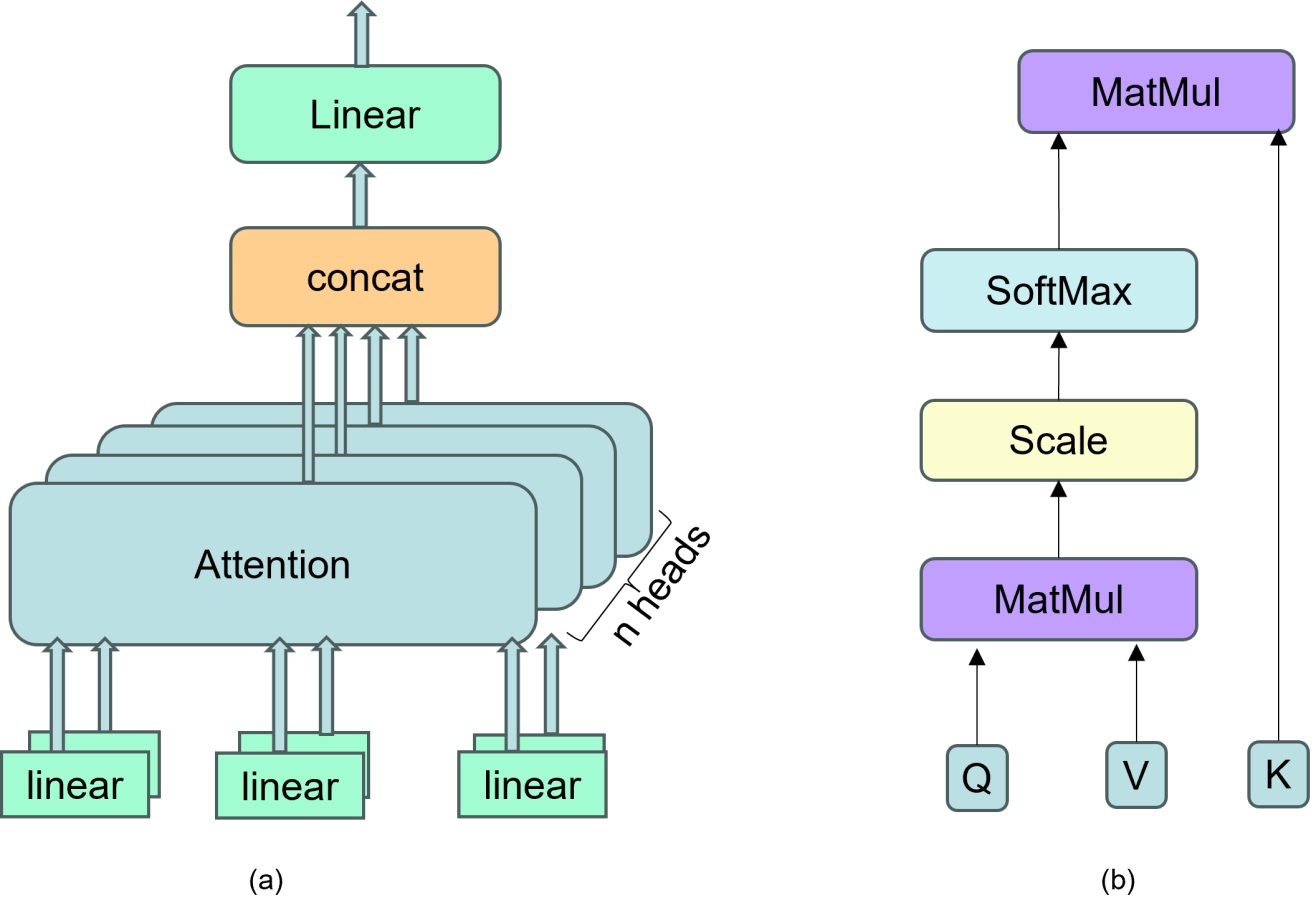
In (**a**), we did not use a fixed-dimension single attention function. Instead, we applied linear projections to the queries, keys, and values and performed parallel attention functions, concatenated the outputs, and then projected them to obtain the final values. In (**b**), the specific attention mechanism is called scaled dot-product attention. The input consists of queries and keys of dimension dk and values of dimension dv. The dot product between the queries and all keys is computed, and each dot product is divided by $\sqrt{dk}$. The softmax function is then applied to obtain the weights of the values.

In the conducted experiments, it was observed that setting the value of head=8 achieved a desirable trade-off between the inference speed of the model and its performance. The specific structure can be referred to as shown in Figure 6, which provides a detailed description of the implementation of the attention mechanism model.

## 5. Experiment

### 5.1. Experimental Setup

The model used in this study was implemented using PyTorch [34]. The network parameters were set as follows: the number of filters was 64, the number of convolution blocks was 16, the context window size was 16, the feature dimension was 64, the hidden layer dimension was 128, and the segment size was 200. The number of layers in the bidirectional RNN neural network and the number of vibration sensors were varied to investigate their impact on the experimental results. The model was initialized with the specified parameters, and separate tests were conducted using the Adam optimizer [35] and the SGD optimizer [36]. The learning rate was set to $1\times 10^{-3}$ for both optimization methods. In total, 120 training epochs were performed, with early stopping implemented to prevent overfitting. Early stopping was triggered if the model did not show improvement for 10 consecutive epochs. The experiments were conducted on an Intel i7-11700 platform with an NVIDIA Tesla T4 (16 GB) and an NVIDIA Titan X (24 GB) used as the hardware setup.

### 5.2. Evaluation Index

Two commonly used evaluation metrics were employed for the assessment: scale-invariant signal-to-noise ratio (SI-SNR) and signal-to-distortion ratio (SDR) [37,38]. SI-SNR is a metric used to evaluate the performance of speech separation or source separation algorithms. It specifically focuses on the signal-to-noise ratio between the separated audio signal and the original mixed signal. SDR is another metric used to assess the performance of audio separation algorithms, and it focuses on the signal-to-distortion ratio between the separated audio signal and the original signal. We chose SI-SNR because it eliminates the influence of the signal’s amplitude dynamic range compared to SNR. Traditional evaluation metrics like PESQ are applicable for assessing speech signals but may not be suitable for high-frequency drill vibration signals. These metrics are primarily designed to evaluate speech quality and intelligibility and may not capture drill vibration signals’ specific characteristics and nuances. Therefore, we have chosen to focus on SI-SNR and SDR as more appropriate evaluation metrics for our particular application.

(17) $$SI-SNR=10\log_{10}\left(\frac{{∥\hat{s}∥}^{2}}{{∥e∥}^{2}}\right)\;$$

For evaluating the enhanced signal *Y* against the reference clean signal, we first normalize both signals and calculate the difference between them (denoted as *e*).

(18) y=starget+einterf+enoise+eartif

(19) $$\left\{\begin{array}{c} \mathrm{Pinterf}={∥\mathrm{starget}∥}^{2}\\ \mathrm{Pnoise}\;=\;{∥\mathrm{enoise}∥}^{2}\\ \mathrm{Partif}\;=\;{∥\mathrm{eartif}∥}^{2}\\ \mathrm{Ptarget}\;=\;{∥\mathrm{starget}∥}^{2} \end{array}\right.$$

(20) $$SDR=10\log_{10}\left(\frac{\mathrm{Ptarget}}{\mathrm{Pinterf}+\mathrm{Pnoise}+\mathrm{Partif}}\right)$$

To calculate the signal-to-distortion ratio (SDR) between the enhanced speech signal y and the reference clean speech signals, the following steps are typically followed. The enhanced boring vibration signal is decomposed into four mutually orthogonal components: starget represents the target-related component related to the clean signal, einterf represents the interference component, enoise represents the noise component, and eartif represents the artificial noise component.

### 5.3. Details of Experiments

#### 5.3.1. Experiment I

We utilized three neural network architectures: RNN, LSTM, and GRU. We separately applied three different neural network architectures to our model to validate their modeling capabilities for input sequences under different neural network configurations. Regarding the number of vibration sensors, we have configured them as both 2 and 4.

#### 5.3.2. Experiment II

For our model’s Section 4.2, we attempted to model and process the input sequence using RNNs with different depths. The expressive capabilities of models with varying depths differ, leading to different modeling abilities for the input sequence. We experimented with seven different depths, ranging from 2 to 8.

#### 5.3.3. Experiment III

To validate the effectiveness of the attention mechanism in our model, we conducted two sets of experiments. One set of experiments used two vibration sensors, while the other used four. In both experiments, the RNN depth was set to 4, and we employed the GRU neural network. The “Original” group represents the model without the attention mechanism module, while the “MSAM 4+” group represents the model with the attention mechanism module.

#### 5.3.4. Experiment IV

To validate the denoising performance of the model in real-world outdoor environments, we conducted experiments using field data collected from Shunyi District, Beijing. In the experiment, the MultiSAMS model utilized a GRU neural network with a depth of 4 and applied denoising and separation operations. To assess the impact of the number of vibration sensors on signal separation, we conducted separate experiments using four-channel and two-channel data and compared their experimental results. Due to the absence of clean signals, we could not obtain SI-SNR and SDR values, so we opted to present the experimental results using spectrograms.

#### 5.3.5. Experiment V

To compare the recognition rates of the MultiSAMS model using different neural networks and recognition models, we conducted experiments with two distinct recognition models and two other neural networks. We validated their recognition performance under varying signal-to-noise ratios (SNR). To compare the effectiveness of single-channel and multi-channel methods, we used the BL-RNN network for single-channel denoising enhancement. Then, we fed the enhanced audio into the recognition model for identification.

## 6. Results

### 6.1. Results and Analysis of Experiment I

Table 1 displays the experimental results obtained using three different neural networks with two vibration sensors. Table 2 presents the experimental results obtained using the same three neural networks with four vibration sensors.

We can observe that, under the same number of vibration sensors, the experimental results using the GRU neural network are superior to those using LSTM and RNN neural networks. Although it may not have the smallest model size, considering the overall experimental results, using the GRU neural network appears to be the optimal choice. When comparing different numbers of vibration sensors, the results with four vibration sensors are significantly better than those with two sensors, especially in terms of SDR, where there is a substantial improvement.

### 6.2. Results and Analysis of Experiment II

From Table 3, the model’s performance was tested with different numbers of GRU layers. It was observed that increasing the number of GRU layers did not necessarily lead to a proportional improvement in separation performance. The experiments were conducted up to eight layers, but it was found that increasing beyond this point resulted in a significant decrease in inference speed and higher hardware requirements without any noticeable improvement in performance. Therefore, it was concluded that the optimal number of GRU layers for the model was four.

### 6.3. Results and Analysis of Experiment III

From Table 4 and Table 5, we can observe that whether using four vibration sensors to receive drilling vibration signals or using two sensors to receive tunneling vibration signals, the use of the attention mechanism module consistently yields better results compared to not using the attention mechanism module. There is a noticeable improvement in both SI-SNR and SDR evaluation metrics.

**Figure 7:**
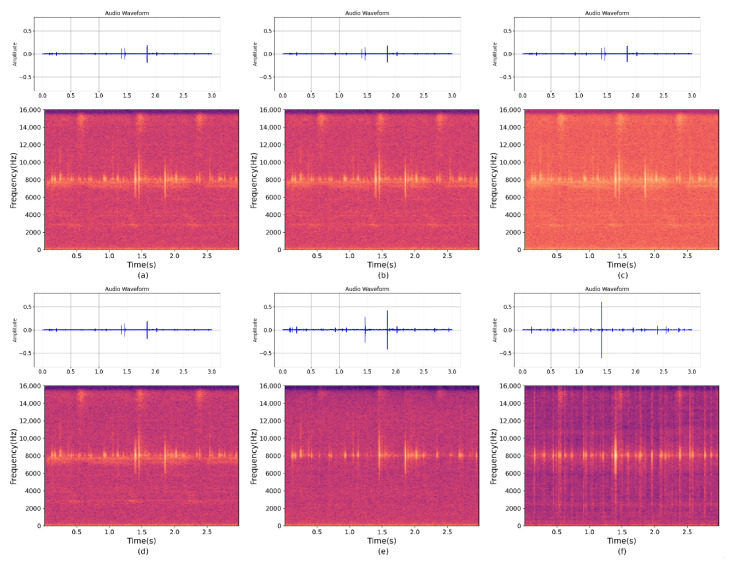
(**a**–**d**) represent the wood-boring vibration signals collected with noise during the same time period. (**e**,**f**) represent two enhanced wood-boring vibration signals separated after being processed by the MultiSAMS model.

**Figure 8:**
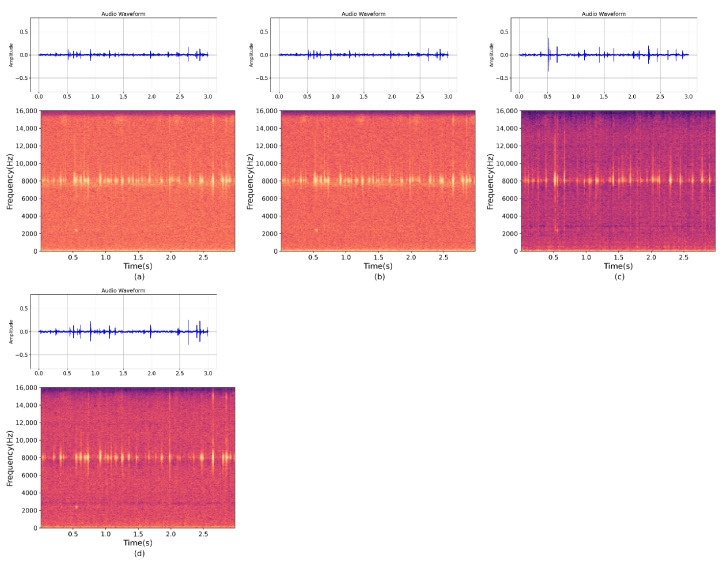
(**a**,**b**) represent the frequency spectrum plots of the wood-boring vibration signals collected by two vibration sensors with noise during the same time period, and (**c**,**d**) represent the frequency spectrum plots of the two clean wood-boring vibration signals separated after being processed by the MultiSAMS model.

As shown in Figure 7 and Figure 8, it presents the frequency spectrum plots of the wood-boring vibration signals collected from different microphone configurations with noise, as well as the frequency spectrum plots after denoising and separation using the MultiSAMS network during the same period. The denoising and separation effect is significantly improved after applying this model.

### 6.4. Results and Analysis of Experiment IV

**Figure 9:**
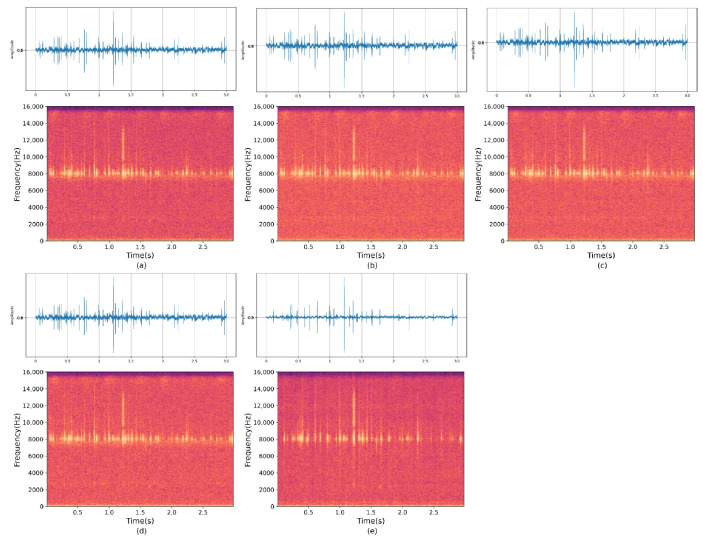
The figure displays the separation results using the MultiSAMS model on data collected in a real-world outdoor environment. (**a**–**d**) represent the spectrograms of the signals from the four channels fed into the model, and (**e**) represents the spectrogram of the separation result.

**Figure 10:**
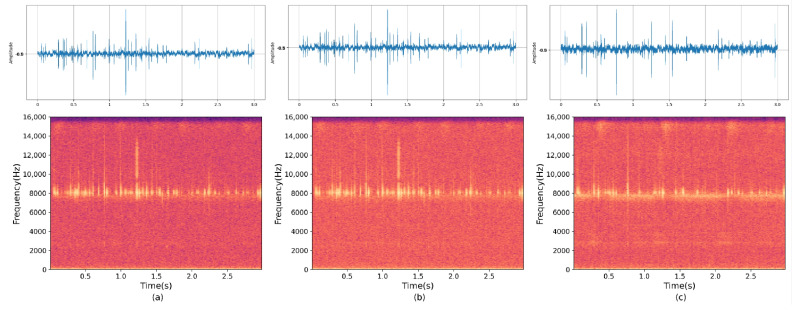
The figure displays the results obtained by feeding the dataset collected in a real-world outdoor environment into the MultiSAMS model and obtaining spectrograms after separation. In this figure, (**a**,**b**) represent the spectrograms of the signals from two channels fed into the model, while (**c**) represents the spectrogram of the signal obtained after separation by the model.

We selected infected white ash trees from Shunyi District, Beijing, to validate the model’s performance on raw, unprocessed data collected in a real-world environment. We inserted probes into the tree bark approximately 3–5 cm below the surface to collect the drill cavity vibration signal. We then fed this collected data into the pre-trained MultiSAMS model for separation experiments. The results of the four-channel separation experiment are shown in Figure 9. To assess the impact of the number of channels on the separation results, we used the same data as the four-channel experiment but removed two of the channels, leaving only two channels for input. The results of this experiment are depicted in Figure 10. It is important to note that both experiments utilized the same dataset, with the only difference being the number of channels fed into the model.

### 6.5. Results and Analysis of Experiment V

**Figure 11:**
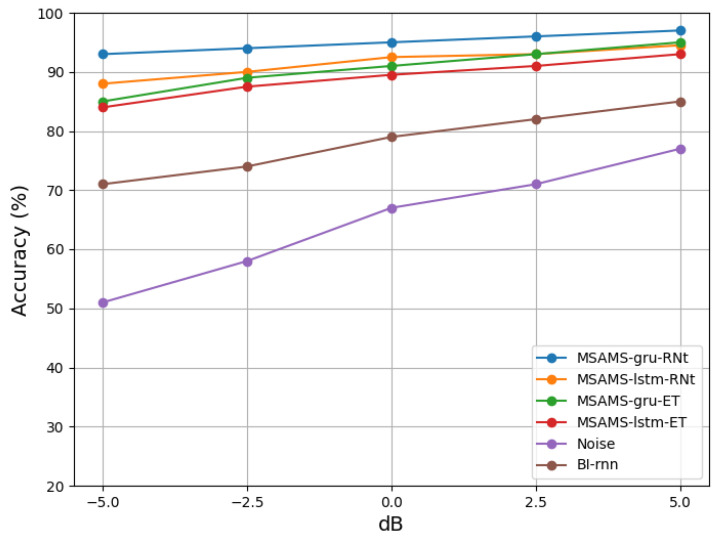
Compared the recognition rates using ResNetSE and ECAPA-TDNN on four different models for noisy segments and enhance segments.

Regarding recognition, we applied two state-of-the-art models with high recognition rates, namely ResNetSE [39] and ECAPA-TDNN [40]. ResNetSE leverages the ResNet architecture and self-attention mechanism to extract highly expressive speech features. On the other hand, ECAPA-TDNN is a speaker verification system incorporating a series of improvements at both the statistical pooling and frame-level layers. These enhancements include channel attention, generalized residual connections, and multi-scale feature extraction. As a result, ECAPA-TDNN achieved outstanding results on multiple benchmark datasets. The specific experimental process involved testing each of the two models independently and evaluating the recognition performance of the BL-RNN model, a time-domain single-channel model with the same structure as the MultiSAMS model and applied on top of ResNetSE. The detailed experimental results are shown in Figure 11. The experiments shown in Figure 11 primarily involve using various configurations of the MultiSAMS model to denoise and separate the input data. Subsequently, the denoised signals are fed into two recognition models to obtain recognition rate results. Figure 11 shows that various models achieve different recognition rates when processing denoised vibration signals. Notably, the MultiSAMS model, which employs GRU neural networks, achieves the highest recognition rate when these denoised signals are subsequently fed into the ResNetSE model. Regarding the recognition models, it is worth noting that the recognition performance of the ResNetSE model is slightly superior to that of ECAPA-TDNN. BL-RNN represents a single-channel processing method using bidirectional recurrent neural networks (RNNs) for denoising enhancement. Based on the experimental results, it is clear that the denoising effectiveness of the MultiSAMS model, regardless of whether it uses GRU or LSTM networks, is superior to the single-channel approach. Furthermore, MSAMS-GRU-RNt achieved the highest recognition performance, slightly surpassing the recognition rate achieved using LSTM neural networks. Table 2 indicates that the parameter count of the GRU neural network is also smaller than that of LSTM, resulting in lower CPU load when using the GRU neural network model.

## 7. Discussion

One relatively simple method for controlling and preventing wood-boring pests is to use acoustic technology to detect problems within tree trunks [4,41]. This involves using embedded piezoelectric accelerometers to measure the vibrations of the trunk and capture these vibrations using vibration sensors. The captured vibration signals are then processed using a multi-channel wood-boring-vibration signal model to detect the presence of pests. In outdoor environments with wood-boring pests, there are typically various noise sources. The presence of environmental noise can indeed disrupt the accurate detection and interpretation of wood-boring-insect signals, leading to false positives or missed detections [42]. Therefore, developing robust multi-channel signal processing techniques and algorithms becomes crucial in mitigating the impact of noise and enhancing the detection of vibrations generated by wood-boring insects.

Our model seamlessly integrates a multi-channel approach for detecting wood-boring vibrations. It incorporates four layers of attention mechanisms, designed to reduce computational complexity through a time-domain-based approach. Nonetheless, including multiple attention layers elevates the model’s complexity, presenting a computational challenge for microcontrollers. Our next phase will prioritize simplifying the model, minimizing computational demands, and enhancing separation performance. There are some limitations in the experimental results. For example, the version of the four-layer model is better than that of the eight-layer model. We suspect this may be due to overfitting and the increased complexity of the network, which could lead to the loss of features during the training process. We will collect more field data and further optimize our model by deploying our microcontrollers in the field. We have only tested the model with two and four vibration sensors and have yet to explore higher numbers. In the future, once we address the hardware limitations, we plan to experiment with a more significant number of vibration sensors to enhance the performance of our model further. In the network section, as depicted in Figure 11, it is evident that GRU networks yield significantly better results than LSTM neural networks. GRU networks are relatively more straightforward in structure than LSTM networks, making them more suitable for low-computational-power microcontroller systems. Our future research will primarily focus on utilizing the GRU network within the MultiSAMS model. We agree that our model can be extended to detect other wood-boring pests, not just limited to the emerald ash borer (EAB). By adapting and fine-tuning the model for different species, we can also apply it to detecting other wood-boring insects. Currently, our research scope has expanded beyond the identification of the white wax narrow beetle. We have also researched the detection and identification of another type of harmful wood-boring insect, the wood-borer moth. We have collected a dataset of wood-borer-moth drilling-vibration signals with a duration exceeding 50 h and have conducted experiments. Through training on the wood-borer moth dataset, MultiSAMS has achieved a recognition rate of over 90% for wood-borer moths. At present, automatic species recognition technology has not been implemented. Whether it is wood-borer moths or white wax narrow beetles, species recognition requires training on the corresponding dataset to achieve recognition of the species. In the future, we will focus on enhancing the model’s ability to recognize different species, aiming to improve the accuracy of species classification recognition. Population recognition will also be a direction for our future research. This would broaden the application and impact of our model in pest detection and management. Expanding the dataset to include a variety of wood-boring pest species and different environmental conditions is a valuable approach to enhance the applicability of our model. By training the model with a diverse range of wood-boring-insect vibration signals, we can improve its ability to detect and classify different pest species. This expanded model can then be used for early warning systems, enabling the implementation of various pest management strategies such as biological control or chemical treatments to minimize the damage caused by wood-boring pests to trees.

## 8. Conclusions

This paper proposes a time-domain-based multi-channel attention mechanism separation model that extracts data and channel-specific features. By fusing these features and feeding them into the attention mechanism, the model can dynamically adjust the weights of different channels and data features in real time, enhancing the retention of relevant information. Experimental results demonstrate that the system effectively removes noise from wood-boring vibration signals and separates clean vibration signals. Detecting wood-boring vibration signals enables efficient control measures for the emerald ash borer (EAB). This significantly improves control effectiveness while reducing costs. Currently, the system is only applied to control the emerald ash borer species. Still, it can be expanded to handle other species by training with more diverse datasets in the future.

## Figures and Tables

**Table 1:** The table below illustrates the performance metrics of the model using three different networks (GRU, LSTM, RNN) when tested with 2 vibration sensors, where Of params is the parameter number and refers to the total number of parameters that must be trained in model training and is used to measure the size of the model (computational space complexity). Sensors represent the number of vibration sensors used in the experiment.

Model	Sensors	Of Params	SI-SNR	SDR
GRU	2	2.5 m	9.27	2.12
LSTM	2	2.9 m	9.15	2.14
RNN	2	1.7 m	8.29	1.53

**Table 2:** The table below illustrates the performance metrics of the model using three different networks (GRU, LSTM, RNN) when tested on 4 vibration sensors.

Model	Sensors	Of Params	SI-SNR	SDR
GRU	4	2.5 m	12.81	10.97
LSTM	4	2.9 m	10.22	8.40
RNN	4	1.7 m	8.75	7.21

**Table 3:** The model’s performance was tested using attention layers ranging from 2 to 8. The model was trained for 120 epochs, and if there was no improvement in performance for 10 consecutive epochs, the training was terminated.

Mode Layer	Of Params	SI-SNR	SDR
2-layer	1.7 m	8.64	6.97
3-layer	2.26 m	9.26	7.53
4-layer	3.1 m	12.81	10.97
5-layer	3.6 m	8.98	7.40
6-layer	4.2 m	7.73	6.21
7-layer	4.8 m	8.34	6.83
8-layer	5.6 m	9.28	7.65

Using 4 sensors, the model receives the vibration signals from the drilling columns.

**Table 4:** The table below shows the comparison experiment between using a multi-head self-attention module (MSAM) with 4 layers and not using MSAM for the case of 4 vibration sensors receiving the drilling vibration signals, i.e., using 4-channel vibration sensors.

Model	Of Params	SI-SNR	SDR
MSAM 4+	2.9 m	13.11	11.09
Original	3.1 m	10.53	8.77

4-channel vibration sensors.

**Table 5:** The table below shows the comparison experiment between using a multi-head self-attention module (MSAM) with 4 layers and not using MSAM for the case of 2 vibration sensors receiving the drilling vibration signals, i.e., using 2-channel vibration sensors.

Model	Of Params	SI-SNR	SDR
MSAM 4+	2.5 m	11.95	10.90
Original	2.4 m	9.17	2.22

2-channel vibration sensors.

## Data Availability

The data presented in this study are available on request from the corresponding author.

## Supplementary Materials

The following supporting information can be downloaded at: https://www.mdpi.com/article/10.3390/insects14100817/s1.
