# Supplementary material for: Multi-Channel Time-Domain Boring-Vibration-Enhancement Method Using RNN Networks
Source: Insects. 2023 Oct 16;14(10):817. doi: 10.3390/insects14100817 (PMC10607493; doi:10.3390/insects14100817)
Supplement: Supplementary file 1 [file insects-14-00817-s001.zip › Audio_data-explain.pdf]

AudioData

Regarding the "Train-data" folder

The data in the "Train-data" folder and the data in the "Figure 7" and "Figure 8" folders have the same source. This is a folder containing a training dataset of 20 examples, using two vibration sensors as an example.

RecordX-clean.wav: It represents the clean drilling vibration signal.The clean drilling vibration signals were collected in a quiet lab using data acquisition boards. For this portion, we performed preprocessing operations to remove segments that did not contain drilling vibration signals.For every 10-15 minutes of recording, a valid 1 minute audio file containing a clean drill string vibration signal can be clipped.

RecordX-mix.wav: It represents the mixed drilling vibration signal with added noise, which is used for model inference and training.

background_noise.wav:These represent noise files added during the synthesis of the dataset. These noise files were recorded under field conditions.

Regarding the "Figure 7" folder and the "Figure 8" folder.

(1).**figureX_X_denoised.wav**:Representing files that have undergone model-based denoising and enhancement processing.For example, "figure7_e_denoised.wav" represents the audio file corresponding to Figure 7, the "e" spectrogram, and it is the audio file after denoising.

(2).**figureX_X_Record.wav**: It is the mixed audio file without model processing.All the audio files are synthesized by adding noise to clean drilling vibration signals.

(3).**background_noise.wav**:These represent noise files added during the synthesis of the dataset. These noise files were recorded under field conditions.

Regarding the "Figure 9" folder and the "Figure 10" folder

(1).**figureX_X_denoised.wav**:Representing files that have undergone model-based denoising and enhancement processing.For example, "figure9_e_denoised.wav" represents the audio file corresponding to Figure 9, the "e" spectrogram, and it is the audio file after denoising.

(2).**figureX_X_RecordX.wav**:It is the mixed audio file without model processing.All the audio files are recorded from real outdoor environmental data without any additional processing. We

extracted audio files containing valid drilling vibration signals from over 50 hours of recorded audio, processed them into 3-second segments, and input them into the model for processing.
